# Comparison of ante- and postmortem ventricular wall thickness using echocardiography and autopsy findings

**DOI:** 10.1007/s00428-024-03960-z

**Published:** 2024-11-08

**Authors:** L. Lohner, B. Ondruschka, J. Garland, R. Tse, A. I. Suling, C. Sinning

**Affiliations:** 1 Institute of Legal Medicine, University Medical Center Hamburg-Eppendorf, Hamburg, Germany; 2 Institute of Medical Biometry and Epidemiology, University Medical Center Hamburg-Eppendorf, Hamburg, Germany; 3 University Heart Center, University Medical Center Hamburg-Eppendorf, Hamburg, Germany; 4 Queensland Public Health and Scientific Services, Coopers Plains, QLD Australia; 5 Griffith University School of Medicine, Southport, QLD Australia

**Keywords:** Cardiac hypertrophy, Ventricular wall thickness, Cardiac dissection, Forensic autopsy, Cardiac pathologies

## Abstract

In autopsy practice, the thickness of ventricular walls is one of the parameters used to identify cardiac hypertrophy. The presented study aimed to compare ante- and postmortem measurements of ventricular wall thickness, (i) to determine a postmortem standardized localization and dissection method for ventricular wall measurements, and (ii) to determine the ability of postmortem measurements in recognition of antemortem hypertrophy. A single-center prospective study was conducted at the Institute of Legal Medicine in Hamburg, Germany. Sixty hearts were dissected alternating by the inflow-outflow or short-axis method, and the ventricular walls were measured at different locations and compared with the echocardiographic values of the end-diastolic phase during life of these individuals. The results showed measurement differences between the autoptic and echocardiographic values—for the left ventricle between 3.3 and 5.2 mm, for the right ventricle between 0.2 and 1.1 mm, and for the septum between 1.3 and 1.4 mm. Diagnostic performance of recognizing antemortem hypertrophy with postmortem measurement was poor, except for measuring the right ventricle and septum with the short-axis method (area under the ROC curve of 0.72 and 0.82, respectively). According to the results, cardiac changes may occur postmortem and need to be considered when used for diagnosing cardiac pathology. The postmortem diagnosis of left or right ventricular hypertrophy should always be made in conjunction with other, particularly cardiac, autopsy findings. An autoptic diagnosis of hypertrophy solely by a ventricular wall thickness > 15 mm or > 5 mm alone is not sufficient.

## Introduction

Sudden cardiac death (SCD) remains one of the most common causes of death and is currently reported as the third leading cause of death in Europe [1, 2]. Against this background, the diagnosis of sudden cardiac death is mainly made for individuals who were previously considered healthy or who had underlying disease but the sudden deterioration resulting in death was unexpected [3]. In autopsy practice, the most common parameters for the objective description of cardiac pathology, in addition to the morphology of myocardial changes, histological and immunohistochemical examinations, are the recording of total heart weight, the measurement of valve circumferences, and in particular the ventricular wall thickness after standardized dissection—either according to the short-axis or the inflow-outflow method [3, 4, 8]. Left ventricular hypertrophy may be associated with a high risk of sudden cardiac death [23, 24].

Antemortem, comparable cardiac measurements are routinely determined by echocardiographic examination [6, 21]. In autoptic and cardiological practice, the measurements of ventricular wall thickness are compared with standard values to detect cardiac hypertrophy [6, 18–20]. These standard values are listed in Table 1. The literature contains various statements on the optimal and “correct” postmortem measurement localization of the ventricular walls. However, a standardized localization for a precise measurement based on studies has not yet been described. According to Sandritter et al. and Sommer et al. the wall thickness of the right ventricle should be measured 1 cm below the pulmonary artery or pulmonary valve, and that of the left ventricle 1 cm below the aortic valve during dissection using the inflow-outflow method [9, 10], whereas Titus et al. recommend measuring the ventricular wall thickness of the left and right ventricle 1 cm below the mitral and pulmonary valves [11]. Sheaff et al. consider the measurement 1 cm below the atrioventricular valves to be useful [12]. Using the short-axis method, Basso et al. recommend measurement in the mid-valve plane between the base and apex of the heart [3, 4].

**Table 1:** Echocardiographic and autoptic standard values for ventricular wall thickness of left and right ventricle, and septum

	Sex	Echocardiography [6]	Autopsy [18–20]
Left ventricle/septum (cm)	Female	0.6–0.9 normal range 1.0–1.2 mildly pathological 1.3–1.5 moderately pathological > 1.5 severely pathological	1.2–1.5 normal > 1.5 pathological
	Male	0.6–1.0 normal range 1.1–1.3 mildly pathological 1.4–1.6 moderately pathological > 1.6 severely pathological	
Right ventricle (cm)	Female /male	0.1–0.5 normal range > 0.5 pathological	0.3–0.5 normal > 0.5 pathological

It should be discussed that the “tilted” incision in the direction of the aortic valve used in the inflow-outflow method can lead to overestimation of the thickness of the left ventricular wall. A potentially useful and comparative method for the designation of a standardized postmortem measurement localization could be the comparison of the postmortem measurement values with those of the antemortem echocardiography. As early as in the 1980s and 1990s, studies were published comparing ante- and postmortem ventricular wall measurements and heart mass [13–17]. However, some of these studies were performed on formalin-fixed hearts and in low-number cohorts. The postmortem ventricular wall thickness correlated poorly with the echocardiographically measured diastolic values and higher with the systolic values [13, 16], while others described diverging results [14]. In a study by Ampanozi et al. and Chatzaraki et al. the measured ventricular wall thicknesses in postmortem magnetic resonance imaging (PMMR) were compared with those measured by gross examination. Again, a significant difference between the values was found [22, 25]. Hypothetically, the ventricular wall thicknesses should be reproducibly measurable by echocardiography and autopsy assuming solid muscle tissue of the same individual. However, experience has shown that the values measured during life and postmortem can differ, as they are based on different and previously uncoordinated measurement methods. The heart can undergo structural changes postmortem and is being compared to dynamic muscle in life that is functioning under pressure. In some cases, this results in different measurement findings when comparing the autopsy to the echocardiography performed. In medicolegal (and also cardiological) practice, this is relevant for expert opinions especially when potential care and treatment concerns for medical care are raised.

The study presented here aimed to compare ante- and postmortem measurements of ventricular wall thickness to determine.

(i) standard localization and dissection method for the measurements of the ventricular walls, and
(ii) the ability of postmortem measurements to recognize antemortem hypertrophy.

## Material and methods

A single-center prospective study was conducted at the Institute of Legal Medicine of the University Medical Center Hamburg-Eppendorf, Hamburg, Germany, in cooperation with the University Heart and Vascular Center of the University Medical Center Hamburg-Eppendorf, from 01/01/2022 to 11/30/2023. Decedents over 18 years of age who had undergone an echocardiographic examination at the University Medical Center Hamburg-Eppendorf up to 6 months before death were included in the study.

Postmortem case files were consequently screened for any information on cardiological investigations prior to death. Exclusion criteria were suspicious deaths due to legal restrictions and decedents with an artificial or biological heart valve replacement, ruptured myocardial infarctions, recent cardiac surgery, moderate-to-severe decomposition/putrefaction, and after extracorporeal membrane oxygenation (ECMO) as well as polytrauma (due to the risk of heart injury).

**Fig. 1:**
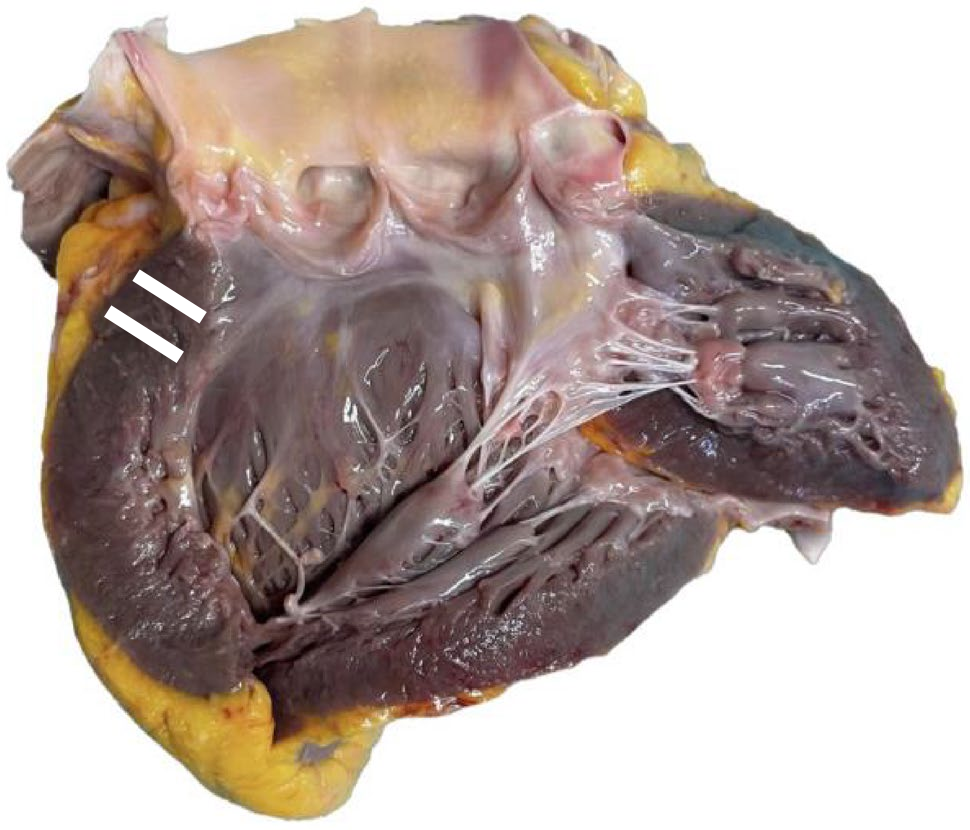
Heart dissection according to the inflow-outflow method. The ventricular walls were measured 1 and 2 cm below the tricuspid valve (for the right ventricle), the aortic valve, and the mitral valve (for the left ventricle). The measurement taken below the aortic valve is illustrated here as an example (indicated white lines)

**Fig. 2:**
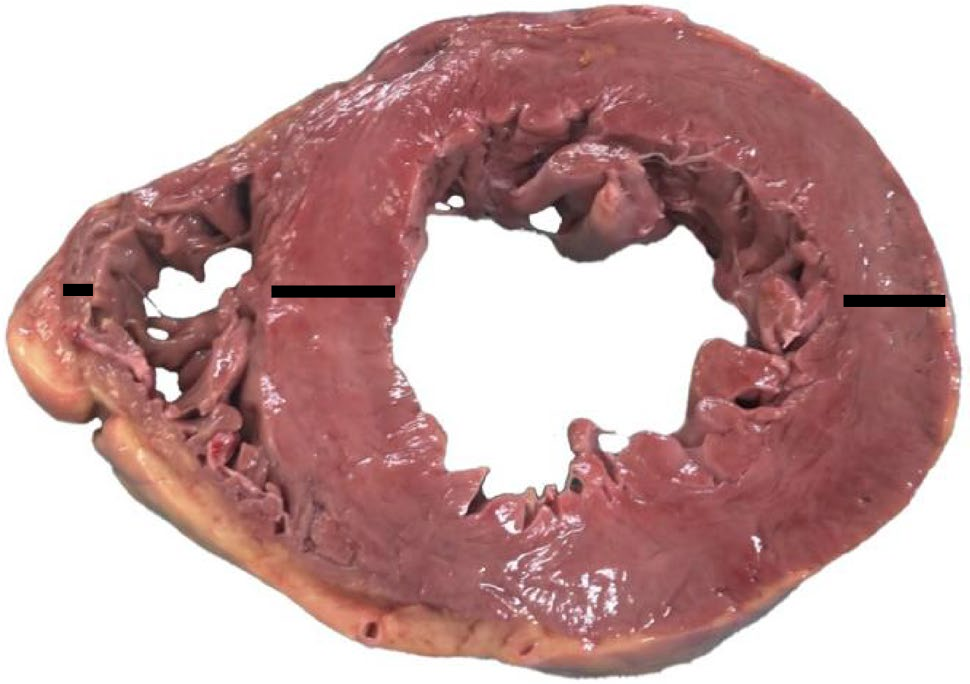
Heart dissection according to the short-axis method. Both the left and right ventricular wall as well as the septum were measured at the level of the papillary muscles (middle ventricular level) on the opposite sides, excluding epicardial fatty tissue and the papillary muscle (black lines)

The study protocol was approved by the Ethics Committee of the Hamburg Medical Association (Application Number 2020–10311-BO-ff). After consent by the next of kin, a partial autopsy was performed with examination of the thoracic cavity and evisceration of the heart from the pericardial sac. The aorta and pulmonary vessels were cut 2 cm above the semilunar valve. Dissection was performed according to the European and American guidelines [3, 5] using either the inflow-outflow (*n* = 30) or the short-axis (*n* = 30) method in an alternating order. In the inflow-outflow method, the right atrium and ventricle were opened and the pulmonary outflow tract was dissected according to the direction of blood flow. Then, the left atrium and left ventricle were opened towards the apex of the heart and back across the aorta. In the short-axis method, a transverse incision was made at the level of the mid ventricle with further parallel transverse slices at 1-cm intervals towards the apex. The basal rest of the heart was dissected in the direction of blood flow. Heart weight, including epicardial fat, was determined after rinsing and drying using the bench scale KERN FCF 30 K-3 (Kern und Sohn GmbH Balingen, Germany) [7]. The thickness of the ventricular walls was measured after complete dissection and before dissection of the coronary arteries by the first author. After dissection using the inflow-outflow method, the ventricular walls were measured twice, 1 and 2 cm below the tricuspid valve, the aortic valve, and the mitral valve using an angle ruler. This was positioned at a 90° angle to the ventricular wall (Fig. 1). A measurement below the pulmonary valve was deliberately omitted because author’s experience and initial measurements at this point showed a very blurred boundary between the fatty tissue and musculature, causing the measurement to be inaccurate. After dissection using the short-axis method, the ventricular walls and the septum were measured at the level of the papillary muscles (mid-ventricular level) on the opposite sides, excluding epicardial fatty tissue and the papillary muscle (Fig. 2). The last author used the available antemortem echocardiographic images to take measurements of the ventricular walls and assessed the ultrasound quality with the grades “very good” to “sufficient.” The cardiologic measurement of the ventricular walls was performed in the two-dimensional ultrasound image end-diastolic for the left ventricle in parasternal ultrasound windows and for the free wall of the right ventricle in the subcostal acoustic window. In the parasternal ultrasound windows, the left ventricular posterior wall and the septum were measured in the parasternal long axis (longitudinal section) and parasternal short axis (transverse section) at the level of the closed mitral valve leaflets [6]. The parasternal long-axis level can be considered similar to the autoptic inflow-outflow method and the short-axis level to the short-axis method. In addition, sex, age, postmortem interval (PMI, as the time interval between death and dissection), body length and weight, and body mass index (BMI) were determined.

### Statistical analysis

Statistical analysis was performed using IBM SPSS Statistics version 29.0 (IBM, Armonk, NY, USA) and Stata 17.0 (StataCorp. 2021. Stata Statistical Software: Release 17. College Station, TX: StataCorp LLC).

The paired *t*-test was performed to compare the measured values of the ante- and postmortem ventricular wall thicknesses, mean difference together with 95% confidence interval (CI), and *p*-value are presented. Pearson’s correlation coefficient was used to check a linear relationship between measurements and agreement was assessed using the intraclass correlation (ICC) coefficient. Furthermore, Bland–Altman plots were used to illustrate agreement between ante- and postmortem measurements. For this, the mean of each pair of measurements was plotted against the difference between them together with the overall mean difference and the respective limits of agreement. The normal distribution was checked visually by QQ plots. Pearson’s correlation coefficient was used to determine correlation between ante- and postmortem measurement differences and PMI. To determine the ability of postmortem measurements in recognition of cardiac hypertrophy that was present during the patient’s lifetime ROC analysis was used. The gold standard was defined as the measurement of hypertrophy on echocardiography before death. The threshold value for hypertrophy during life was set at 13 mm for the left ventricle and the septum, and 5 mm for the right ventricle according to the literature [6]. This was compared with hypertrophy diagnosed with postmortem measurements based on thresholds presented in the literature: a ventricular wall thickness of 15 mm and more for the left ventricle or septum and 5 mm or more for the right ventricle was considered the threshold value for hypertrophy [18–20]. Also, an optimal threshold was estimated using the Liu method (27) or defined according to clinical relevance. The area under the ROC curve and sensitivity, specificity, positive predictive value (PPV), as well as negative predictive value (NPV) are presented for those thresholds.

## Results

### Demographic data

In the presented study, 60 decedents with a mean postmortem interval (PMI) of 2.9 days (SD 2.4, median 2.1, range 0.4–14.6) and a mean age of 72.3 years (SD 16.1, median 77.0, range 29–100 years) underwent partial autopsy with heart dissection, and the thickness of the ventricular walls was measured as introduced above. The sex ratio was 29:31 (f:m). The average postmortem body weight was 76.5 kg (SD 25.2, median 68.9, range 38.5–144.2), and the average body length was 170.1 cm (SD 12.0, median 169.5, range 140.0–196.0). The average BMI was 26.1 kg/m^2^ (SD 6.7, median 24.3 kg/m^2^, range 14.6–43.3). The interval between the echocardiographic examination and death or the time of dissection was on average 31.8 or 34.4 days respectively (SD 39.4 resp. 39.1, median 12.5 resp. 15.0, range 0.0–165.0 resp. 3.0–168.0). The average heart weight after dissection, rinsing, and drying was 488.6 g (SD 167.2, median 442.5, range 210–1016). In eight out of 60 decedents, sepsis was present at the time of death due to pneumonia or immunosuppression in leukemia according to the clinical records. The quality of echocardiography was reported as “very good” in 11 cases, as “good” and “adequate” in 18 cases, and as “satisfactory” in 13 cases (average 2.6, SD 1.1, median 3.0).

### Comparison of the echocardiographic and autopsy measurements

**Fig. 3:**
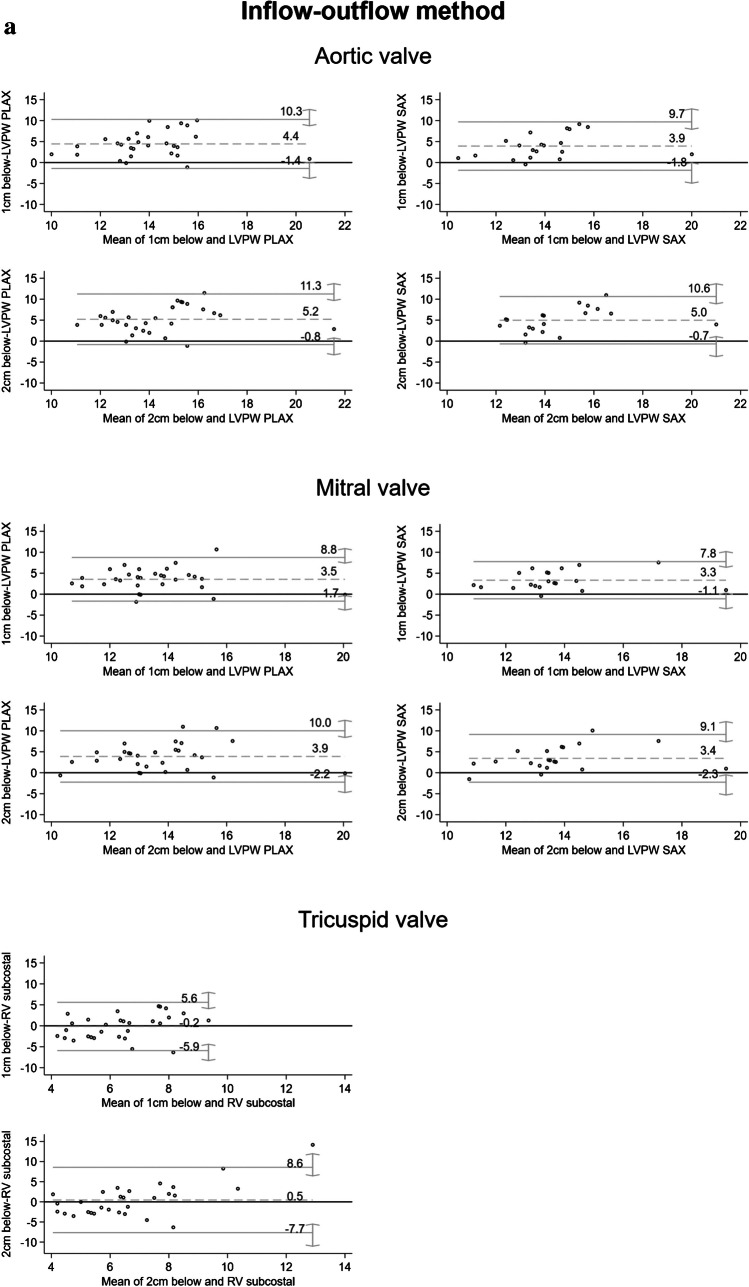

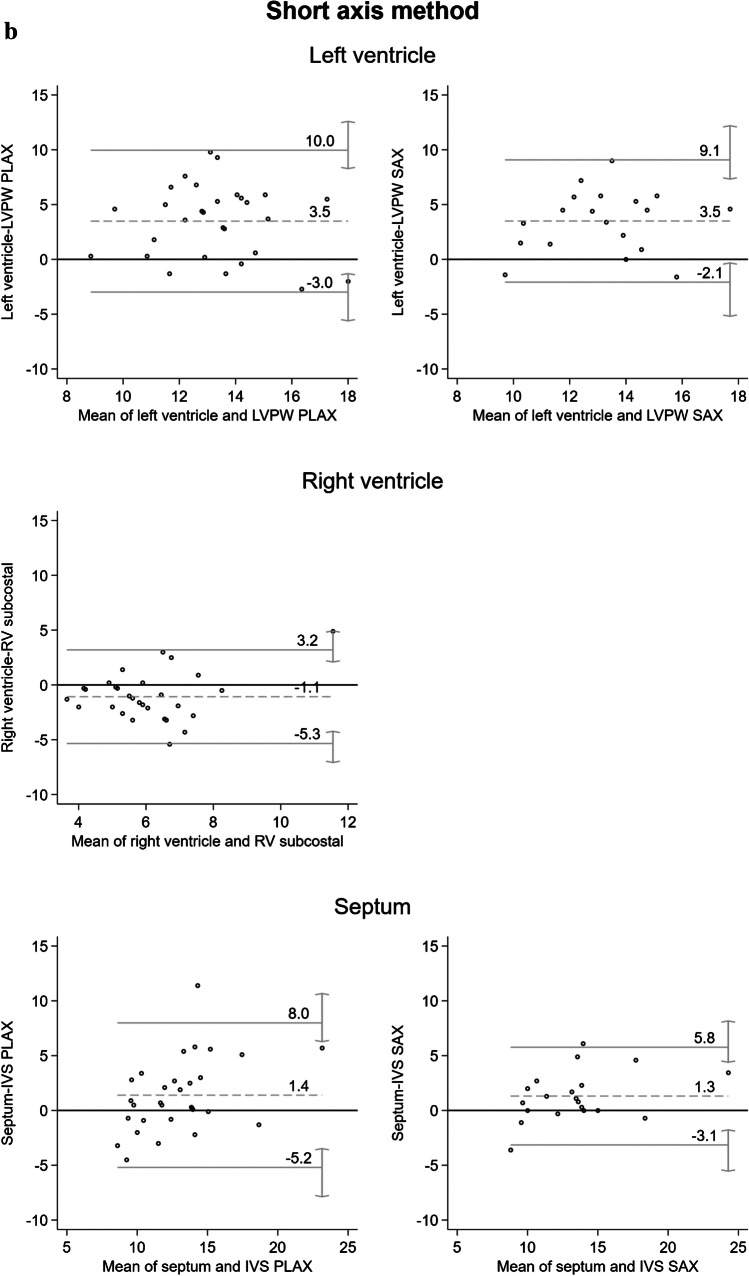
**a** Bland–Altman plots illustrating the differences between the measurements against their mean values for the inflow-outflow method for all measured localizations. LVPW PLAX, left ventricular posterior wall thickness in parasternal long axis; LVPW SAX, left ventricular posterior wall thickness in short axis; RV subcostal, right ventricular wall thickness subcostal. **b** Bland–Altman plots illustrating the differences between the measurements against their mean values for the short-axis method are shown in the figure for all measured localizations. RV subcostal*,* right ventricular wall thickness subcostal; IVS PLAX*,* interventricular septum thickness in parasternal long axis; IVS SAX*,* interventricular septum thickness in short axis

The echocardiographic and autopsy measurements were compared using the paired *t*-test. In addition, Pearson’s correlation coefficient and the intraclass correlation (ICC) were calculated. The results are shown in detail in Table 2. The Bland–Altman plots do not show a high level of agreement between the echocardiographic and the autoptic measurements (Fig. 3a and b). Deviations up to 10 mm could be detected when measuring the left ventricle. The largest differences were found for the measurements of the left ventricle using both dissection methods with the ventricle wall thickness being higher in the postmortem measurement.

**Table 2:** Comparison of the echocardiographic and autoptic measurements for the left and the right ventricle, and the septum

Localization of autoptic measurements	Autoptic measurement MV (± SD) [mm]	Echocardiographic level	Echocardiographic measurement MV (± SD) [mm]	Difference in values^a^ MV [95%-KI]	*p*-value (two-sided)	Pearson correlation^b^	ICC
Inflow-outflow method							
1 cm below the aortic valve	16.2 (± 2.6)	LVPW PLAX	11.8 (± 2.3)	4.4 [3.3; 5.5]	< 0.001	0.25	0.09
	15.9 (± 2.8)	LVPW SAX^1^	12.0 (± 2.1)	3.9 [2.6; 5.3]	< 0.001	0.28	0.12
2 cm below the aortic valve	17.0 (± 2.8)	LVPW PLAX	11.8 (± 2.3)	5.2 [4.1; 6.4]	< 0.001	0.28	0.09
	17.0 (± 2.9)	LVPW SAX	12.0 (± 2.1)	5.0 [3.6; 6.3]	< 0.001	0.38	0.12
1 cm below the mitral valve	15.3 (± 2.2)	LVPW PLAX	11.8 (± 2.3)	3.5 [2.6; 4.5]	< 0.001	0.29	0.13
	15.3 (± 2.3)	LVPW SAX	12.0 (± 2.1)	3.3 [2.3; 4.4]	< 0.001	0.48	0.22
2 cm below the mitral valve	15.6 (± 2.5)	LVPW PLAX	11.8 (± 2.3)	3.9 [2.7; 5.1]	< 0.001	0.17	0.07
	15.4 (± 2.8)	LVPW SAX	12.0 (± 2.1)	3.4 [2.0; 4.8]	< 0.001	0.30	0.15
1 cm below the tricuspid valve	6.3 (± 2.3)	RVsubcostal	6.4 (± 1.6)	-0.2 [-1.3; 1.0]	0.778	-0.06	-0.06
2 cm below the tricuspid valve	6.9 (± 3.7)	RVsubcostal	6.4 (± 1.6)	0.5 [-1.1; 2.0]	0.550	-0.05	-0.04
Short-axis method							
Left ventricle	15.0 (± 2.4)	LVPW PLAX	11.5 (± 2.7)	3.5 [2.3; 4.7]	< 0.001	0.19	0.10
	14.9 (± 2.6)	LVPW SAX	11.4 (± 2.3)	3.5 [2.1; 4.9]	< 0.001	0.34	0.17
Right ventricle	5.5 (± 2.1)	RV subcostal	6.6 (± 1.6)	-1.1 [-1.9; -0.3]	0.011	0.33	0.28
Septum	13.6 (± 4.2)	IVS PLAX	12.2 (± 2.8)	1.4 [0.1; 2.7]	0.030	0.59	0.52
	14.0 (± 4.2)	IVS SAX	12.7 (± 3.4)	1.3 [0.2; 2.4]	0.018	0.84	0.78

*ICC* intraclass correlation, *LVPW PLAX* left ventricular posterior wall thickness in parasternal long axis, *LVPW SAX* left ventricular posterior wall thickness in short axis, *RV subcostal* right ventricular wall thickness subcostal, *IVS PLAX* interventricular septum thickness in parasternal long axis, *IVS SAX* interventricular septum thickness in short axis

^1^Echocardiographic measurement in the short axis was not possible in all cases

^a^Paired samples *t*-test

^b^Pearson’s correlation coefficient

The largest measurement differences were found for the measurements below the aortic valve. A measurement of 1 cm and 2 cm below the aortic valve resulted in a difference of 4.4 mm (95% CI [3.3; 5.5]) and 5.2 mm (95% CI [4.1; 6.4]) mm respectively between the echocardiographic (parasternal long axis) and the autoptic measurement. The differences for the measurements below the mitral valve are somewhat smaller—in particular, the measurement 1 cm below the mitral valve showed differences between 3.3 mm (95% CI [2.3; 4.4]) and 3.5 mm (95% CI [2.6; 4.5]) mm, depending on the comparison with the echocardiographic measurement plane. This measurement localization resulted in a positive Pearson correlation with a medium effect size (0.48) and a poor ICC of 0.22. For the right ventricle, slightly lower values were measured for the measurement 1 cm below the tricuspid valve than for the echocardiographic examination (− 0.2 (95% CI [− 1.3; 1.0])), whereas the opposite was true for a measurement 2 cm below the tricuspid valve (0.5 (95% CI [− 1.1; 2.0])). Overall, the comparison of the measurements of the right ventricle showed smaller differences, with small negative correlation coefficients and poor ICC showing poor agreement. In only three out of eight decedents clinically diagnosed with sepsis, measurement differences of up to 11 mm for the left ventricle and septum, and up to 5 mm for the right ventricle were observed.

While the measurement differences after using the short-axis dissection were 3.5 mm (95% CI [2.1; 4.9]) for the left ventricle, the measurements of the septum and the right ventricle showed smaller differences. In addition, Pearson’s correlations for the measurement in the short-axis dissection showed a medium effect size for the left and right ventricle (0.34 and 0.33 respectively), a large effect size for the septum (0.84), and a good agreement (ICC 0.78).

When considering the overall cohort, there was a tendency towards lower measurement differences with increasing PMI (> 2 days) with a small positive correlation for the measurements 1 and 2 cm below the aortic valve (0.25; 0.22) and 1 and 2 cm below the tricuspid valve (0.28; 0.29), and a small negative correlation for the measurements of the left (− 0.13) and right ventricle (− 0.19), and the septum (− 0.31) after using the short-axis dissection.

A further aim of the study was to determine whether postmortem measurements were able to confirm antemortem hypertrophy. The results are presented in Table 3. The diagnostic performance of all postmortem inflow-outflow measurements can be classified as a failure, as the area under the ROC curve is below 0.6 (an area under the ROC curve of 0.5 is equivalent to chance, while 1 indicates perfect discrimination [26]). This is also reflected in a sensitivity between 67 and 76% and a small specificity between 0 and 33%.

**Table 3:** Summarized results of ROC-Analyses

Localization of autoptic measurements	Area under the ROC curve	Threshold value hypertrophy presented in literature / optimal cut-off* [mm]	Sensitivity [%]	Specificity [%]	PPV [%]	NPV [%]
*Inflow-outflow method*						
1 cm below the aortic valve	0.457	≥ 15/ ≥ 21	67/33	19/96	8/50	83/93
2 cm below the aortic valve	0.432	≥ 15/ ≥ 23	67/22	11/100	8/100	75 / 93
1 cm below the mitral valve	0.519	≥ 15/ ≥ 20	67/33	33/96	10/50	90/93
2 cm below the mitral valve	0.494	≥ 15/ ≥ 20	67/33	33/89	10/25	90/92
1 cm below the tricuspid valve	0.488	≥ 5 / ≥ 7	68/44	0/80	77/92	0/22
2 cm below the tricuspid valve	0.524	≥ 5/ ≥ 8	76/36	20/80	83/90	14/20
*Short-axis method*						
Left ventricle	0.558	≥ 15/ ≥ 20**	75/25	35/100	15/100	90/90
Right ventricle	0.720	≥ 5/ ≥ 5	80/80	60/60	91/91	38/38
Septum	0.823	≥ 15/ ≥ 20**	57/29	74/96	40/67	85/81

*PPV* positive predictive value, *NPV* negative predictive value

*Optimal cut-off estimated using the Liu method [27]

**Cut-off defined according to clinical relevance

While measurements with the short-axis method for the left ventricle yielded similar results (sensitivity 75%, specificity 35%), the area under the ROC curve for the right ventricle (0.720) and septum (0.823) shows a fair or considerable diagnostic performance, respectively. Using the threshold for postmortem measurements presented in the literature, for 80% of patients with a hypertrophy diagnosed antemortem in the right ventricle this can also be confirmed postmortem after short-axis dissection (sensitivity 80%), leaving 20% of the patients with a hypertrophy antemortem undiagnosed. Sixty percent of patients without hypertrophy during their lifetime are recognized accordingly postmortem (specificity 60%). The probability of having had hypertrophy during lifetime if diagnosed postmortem using the published thresholds is 91% for the right ventricle, but the probability of not having had hypertrophy if diagnosed as such postmortem is 38%. The test diagnostics when using the septum measurements show opposite directions, with a test being more specific and less sensitive. Seventy-four percent of patients without hypertrophy antemortem are recognized accordingly postmortem, and 57% of patients with hypertrophy during lifetime could be confirmed postmortem after using short-axis dissection. The probability that persons did not have antemortem hypertrophy, when none was detected during autopsy was 85% for the septum and 90% for the left ventricle, while the probability of having had hypertrophy in the patient’s lifetime if diagnosed postmortem is only 15% for the left ventricle and 40% for the septum. Optimal cut-off values calculated in the ROC analysis resulted in postmortem threshold values with higher specificities and PPV (Table 3). Postmortem threshold values between 20 and 23 mm were found for the left ventricle and septum (specificity 89–100%), and between 7 and 8 mm for the right ventricle using the inflow-outflow method (specificity 80%). Using the short-axis method, the threshold given in the literature for the right ventricle could be confirmed.

## Discussion

The postmortem diagnosis of right or left ventricular hypertrophy is often made based on ventricular wall thickness, whereby different measurement localizations are currently given in the literature, which differ somewhat from each other [3, 4, 9–12]. This study shows that echocardiographic and autopsy measurements of ventricular wall thickness differ and are not to be expected as “equally.” Although standardized procedures were used for the ante- and postmortem measurement, the autoptic measurement cannot be transferred to the clinical measurement and vice versa. The measurement values were determined in the same individuals—ante- and postmortem—by two identical examiners.

The results show mean measurement differences between the ante- and postmortem measurements of 3.3 mm (95% CI [2.3; 4.4]) to 5.2 mm (95% CI [4.1; 6.4]) for the left ventricle and 0.2 mm (95% CI [− 1.3; 1.0]) to 1.1 mm (95% CI [− 1.9; − 0.3]) for the right ventricle for both dissection methods. For the measurement of the septum, the average differences were between 1.3 mm (95% CI [0.2; 2.4]) and 1.4 mm (95% CI [0.1; 2.7]). The highest Pearson correlation was found for the left ventricle measuring 1 cm below the mitral valve (using the inflow-outflow method) compared with the echocardiographic measurement in the parasternal short axis (*p* = 0.48; medium effect size). The measurements below the aortic valve showed higher measurement differences than below the mitral valve with small correlation coefficients. The smallest mean differences were found in the measurements of the right ventricle, 1 cm below the tricuspid valve, and in the short-axis method. Of course, the right ventricle is thinner than the left ventricle and smaller absolute differences are to be expected. For the measurement of the left ventricle and the septum using the short-axis dissection, there were also medium effect sizes of correlations compared to the echocardiographic measurement in the parasternal short axis (*r* = 0.34 and *r* = 0.84, respectively). Reasons for the difference in the values could be the postmortem changes in the heart, possibly due to putrefaction that has already set in [28], water retention due to inflammation and sepsis—although, it certainly depends on the severity of the sepsis, altered wall tension due to lack of blood filling of the heart chambers, or lack of stabilization by the pericardium after dissection. In this study, larger measurement differences were found in a minority of sepsis individuals. In addition, the values of the echocardiographically measured ventricular wall thicknesses in systole can come closer to those measured postmortem assuming muscle contraction and rigor mortis as muscular functions, whereby the heart is measured in the end-diastolic phase during the patient’s lifetime as a standard procedure. The conclusions from the correlations between the PMI and the measurement differences presented here were determined at a cluster level and cannot be used to make statements at the individual level. To determine whether the measurement differences change over time since death within a corpse, several measurements of a heart over a period of time are required as part of a new study.

Considering the cardiologic and autopsy threshold for left ventricular hypertrophy (13 mm vs. 15 mm), the results showed that the diagnostic performance of postmortem measurements for the left ventricle failed after both dissection methods (the area under the ROC curve is below 0.6). Only 8–15% of those diagnosed with postmortem left ventricular hypertrophy had left ventricular hypertrophy during their lifetime, whereas the tests for the right ventricle showed higher PPVs, especially after using short-axis dissection. In contrast to the measurements of the left ventricle and septum, the postmortem diagnosis of hypertrophy in the right ventricle was correct for 77–91% of people, for which it was also present during their lifetime. This is also reflected in the area under the ROC curve, showing a considerable diagnostic performance for the right ventricle (AUC 0.720) and septum (AUC 0.823), respectively. In summary, it was shown that measurement of the right ventricle after short-axis dissection is well suited for the postmortem diagnosis of right ventricular hypertrophy. Ventricular wall thicknesses that exceeded the autopsy limits of 15 mm for the left ventricle or 5 mm for the right ventricle were more specific in finding or confirming a hypertrophy that was present during lifetime, which is why it is recommended that the autopsy limits be adjusted to avoid false diagnoses. However, a larger study cohort would be necessary to define a threshold with optimal diagnostic accuracy (e.g. as part of a multicenter study). Basically, the higher the threshold would be defined, the more accurate postmortem statements will confirm preexisting medical conditions. According to the ROC analysis, higher specificities and PPV were found at higher postmortem threshold values; although, it should be noted that the PPV is prevalence-dependent. So as this cohort consists of patients with a rather high prevalence of cardiovascular diseases, these results may not be transferable to a general population. Nevertheless, it was shown that the threshold values previously reported in the literature are rather too low to diagnose hypertrophy postmortem based on ventricular wall thickness alone. In this study, threshold values between 20 and 23 mm were found for the left ventricle and septum, and values between 5 and 8 mm for the right ventricle after both dissection methods as alternative cut-offs.

In conclusion, we recommend measuring the left ventricular wall thickness 1 cm below the mitral valve and the right ventricular wall thickness 1 cm below the tricuspid valve when using the inflow-outflow method. The results further support the recommendation of Basso et al. to dissect the heart with the short-axis method to detect cardiac hypertrophy [3, 4]. The postmortem diagnosis of hypertrophy should always be made in conjunction with other autopsy findings, especially the cardiac findings. An autoptic diagnosis of hypertrophy solely by ventricular wall thicknesses > 15 mm or > 5 mm alone is not sufficient.

## Limitations

Measurements were mainly carried out on the hearts of deceased persons who already had a history of cardiovascular disease during their lifetime. As the study population included mainly older people, the echocardiographic conditions were not as optimal as for younger people, which could also be a reason for measurement differences. Furthermore, different echocardiographic measured values in systole and diastole resulted, whereby only end-diastolic measured values were included in this study, as only these values are of importance in cardiological practice. On the other hand, measurements of the ventricular walls in autopsy practice are difficult to measure to the millimeter, as the borders of the heart muscle to the epicardial fatty tissue or the papillary muscles can be obscured and the heart no longer has its original shape due to the dissection. Further, different postmortem alterations occur affecting the heart tissue integration after death (rigor, lysis, decomposition), resulting in heart dimension changes within an individual. Here, a single time point of measurement was used after dissection as this reflects the realistic scenario in autopsy practice.

## Data Availability

The datasets generated during and/or analyzed during the current study are available from the corresponding author on reasonable request.
